# Safety and blood levels of daratumumab after switching from intravenous to subcutaneous administration in patients with multiple myeloma

**DOI:** 10.1007/s10637-023-01392-1

**Published:** 2023-09-18

**Authors:** Kenta Yamaoka, Kei Irie, Nobuhiro Hiramoto, Masaki Hirabatake, Hiroaki Ikesue, Tohru Hashida, Tadashi Shimizu, Takayuki Ishikawa, Nobuyuki Muroi

**Affiliations:** 1 Department of Pharmacy, Kobe City Medical Center General Hospital, 2-1-1 Minatojima-Minamimachi, Chuo-ku, Kobe, 650-0047 Japan; 2 School of Pharmacy, Hyogo Medical University, 1-3-6 Minatojima, Chuo-ku, Kobe, 650-8530 Japan; 3 Department of Clinical Pharmacy Research, Center for Clinical Research and Innovation, Kobe City Medical Center General Hospital, 2-1-1 Minatojima-Minamimachi, Chuo-ku, Kobe, 650-0047 Japan; 4 Faculty of Pharmaceutical Science, Kobe Gakuin University, 1-1-3 Minatojima, Chuo-ku, Kobe, 650-8586 Japan; 5 Department of Hematology, Kobe City Medical Center General Hospital, 2-1-1 Minatojima-Minami- machi, Chuo-ku, Kobe, 650-0047 Japan

**Keywords:** Daratumumab, Multiple myeloma, Subcutaneous, Intravenous, Pharmacokinetics

## Abstract

The intravenous administration (IV) of daratumumab sometimes causes an infusion reaction and needs a long infusion time. Recently, a subcutaneous formulation (SC) of daratumumab, which has fewer infusion reactions and shorter administration time, was approved. However, because SC has a fixed dose, overdosing is a concern for patients with low body weights. In this study, we investigated the safety and blood levels of daratumumab after switching from IV to SC in patients with multiple myeloma (MM). Patients who switched from IV to SC of daratumumab between June 2021 and May 2022 at Kobe City Medical Center General Hospital were included in the study. Blood daratumumab levels were measured using liquid chromatography-tandem mass spectrometry. Safety after switching from IV to SC was evaluated for six months and graded according to the Common Terminology Criteria for Adverse Events, version 5.0. The median body weight of ten patients included in the analysis was 57.4 kg (range: 45.0–74.4). Blood daratumumab levels were significantly increased after switching to SC (p = 0.002); median through concentration at the last IV dose was 403.6 μg/mL (range: 96.3–776.3) and that at the third SC dose was 557.1 μg/mL (range: 288.3–997.2). Grade 1–2 injection site reactions were observed in six patients (60.0%) after switching to SC. A new grade 3 adverse event was observed in only one patient (neutropenia). The blood levels of daratumumab were significantly increased after switching from IV to SC in patients with MM; however, the dosage was tolerable.

## Introduction

Daratumumab, a human IgGκ monoclonal antibody that targets CD38, has been demonstrated to have high therapeutic efficacy against multiple myeloma (MM) through complement-dependent cytotoxicity, antibody-dependent cytotoxicity, and antibody-dependent cell phagocytosis [1]. Daratumumab, bortezomib, and dexamethasone combination therapy significantly prolongs the progression-free and overall survival in patients with relapsed and refractory MM [2]. Furthermore, when combined with lenalidomide and dexamethasone, daratumumab prolongs progression-free and overall survival in those patients with newly diagnosed MM who are ineligible for autologous stem cell transplantation [3].

The infusion reaction (IR) is one of serious adverse effects of the intravenous administration (IV) of daratumumab [4]. In the CANDOR trial, despite premedication with acetaminophen and antihistamines, IR occurred in 77% of patients, and grade 3 or more severe symptoms were observed in 28% of patients. Other studies on IV of daratumumab have reported similar IR rates [5]. Therefore, IV of daratumumab is carefully administered at a slow infusion rate, which results in a long infusion time (3–7 h) [6].

Recently, a subcutaneous formulation (SC) of daratumumab was approved for the treatment of MM. The SC of daratumumab contains recombinant human hyaluronidase PH20 (rHuPH20), an enzyme that hydrolyzes hyaluronic acid in the subcutaneous space. This SC has been proved to be equivalent to IV of daratumumab in terms of therapeutic efficacy and pharmacokinetics (PK) [7]. In addition, in COLUMBA study, SC of daratumumab showed a lower incidence of IR (12.7% vs. 34.5%) and shorter administration time (3–5 min vs. 3–7 h) when compared with the IV [7].

The IV dose of daratumumab was specified to be 16 mg/kg of the body weight, whereas the SC dose was fixed at 1800 mg, regardless of the body weight. A fixed SC dose of daratumumab was reported to increase the exposure in patients weighing less than 65 kg, and these patients were more likely to experience myelosuppression [7, 8]. SC of daratumumab is beneficial for both patients and medical staff; however, overdosing is associated with low body weight. In addition, little evidence is available regarding the safety and PK associated with the switching from IV to SC of daratumumab. In the present study, we have evaluated the safety and levels of daratumumab in the blood of patients with MM after switching from IV to SC.

## Materials and methods

### Patients

Patients with MM who switched from IV to SC of daratumumab between June 2021 and May 2022 at Kobe City Medical Center General Hospital (Kobe, Japan) were included in this study. Patients who did not receive at least two SC doses after switching were excluded from the analysis. Written informed consent was obtained from all the study subjects. This study was conducted in accordance with the principles of the Declaration of Helsinki and was approved by the Ethics Committee of Kobe City Medical Center General Hospital (approval number: zn210615; approval date: June 3, 2021).

### Measurement of daratumumab levels in blood samples

**Fig. 1:**
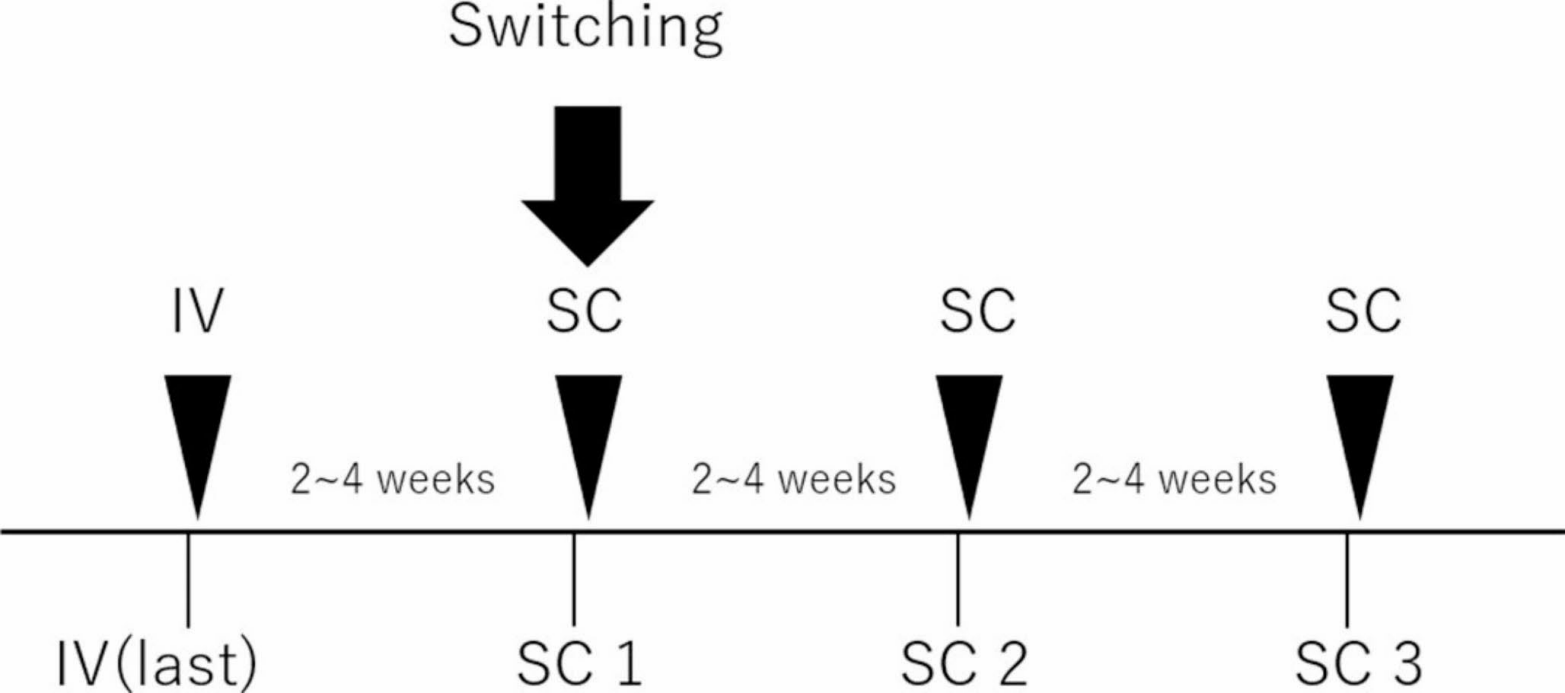
Sampling schedule for the evaluation of blood levels of daratumumab during the switch from intravenous to subcutaneous formulation

The levels of daratumumab in the blood samples were evaluated a total of four times before and after switching from IV to SC, as shown in Fig. 1. IV (last) indicates the blood concentration at the last IV dose before switching to SC. SC1, SC2, and SC3 indicate the concentration of daratumumab in the blood at the first, second, and third SC dose, respectively, after switching from IV to SC. Blood samples were collected before the administration of each dose (trough level). The serum samples were stored at -20 °C and daratumumab levels were measured using liquid chromatography tandem mass spectrometry (LC-MS/MS) as previously reported [9]. Briefly, serum IgG were purified using rProtein A (San Jose, CA)., followed by trypsin digestion, and specific peptides (SNWPPTFGQGTK, m/z: 660.0→932.3) for daratumumab were detected using LC-MS/MS. The calibration range was 50–1000 μg/mL, and the limit of quantification (LOQ) was 50 μg/mL. The assay accuracy (n = 5, relative error) and precision (n = 5, relative standard deviation) were 92.0–113.0% and 2.0–9.4%, respectively. The changes in the levels of daratumumab in the blood samples were evaluated using the Friedman test after Bonferroni correction and were considered significant at p < 0.05.

### Safety evaluation

The safety of daratumumab after switching to SC was evaluated up to six months after the switch because blood levels are expected to be at a steady state within six months. Adverse events (AEs) were graded based on the National Cancer Institute Common Terminology Criteria for Adverse Events (CTCAE) version 5.0.

## Results

### Baseline characteristics of the patients

Twelve patients who switched from IV to SC of daratumumab were included in this study. Two of these patients were excluded from the analysis because daratumumab was discontinued before the administration of two doses of SC. One patient was scheduled for autologous stem cell transplantation, and discontinued SC after one dose. Another patient showed disease progression prior to switching to SC and discontinued the treatment after one dose of SC owing to the disease progression. Thus, 10 patients were assessed in the present study, and their characteristics are shown in Table 1. The median age was 71 years (range, 40–80 years), median weight was 57.4 kg (range, 45.0–74.4 kg), median body mass index (BMI) was 23.3 kg/m^2^ (range, 16.5–25.9), and the type of MM was IgG in eight cases. The treatment regimen used at the time of switching to SC was a combination of daratumumab, lenalidomide, and dexamethasone in seven patients. The daratumumab dosing interval when switching to SC was every four weeks for eight patients and every two weeks for two patients.

**Table 1:** Patient characteristics

Characteristics	Median	Range
Age (year)	71	40–80
Body weight (kg)	57.4	45.0–74.4
Height (cm)	156.3	148.2–173.5-
BMI (kg/m^2^)	23.3	16.5–25.9
eGFR (mL/min/1.73 m^2^)	66	46–86
Total number of doses of daratumumab (times)	27	10–39
Sex (n)		
Male	3	
Female	7	
ECOG PS (n)		
0	6	
1≤	4	
ISS (n)		
I	1	
II	6	
III	3	
Type of multiple myeloma (n)		
IgA	3	
IgG	6	
BJP	1	
Prior lines of therapy (n)		
0	2	
1	3	
2	4	
3≤	1	
Prior stem-cell transplantation (n)	7	
Current therapy (n)		
DLd	7	
DCd	1	
DBd	1	
D-MPB	1	

BMI, Body Mass Index; eGFR, estimated Glomerular Filtration Rate; ECOG PS, Eastern Cooperative Oncology Group Performance Status; ISS, International Staging System; BJP, Bence-Jones Protein; DLd, Daratumumab, Lenalidomide, and Dexamethasone; DCd, Daratumumab, Carfilzomib, and Dexamethasone; DBd, Daratumumab, Bortezomib, and Dexamethasone; D-MPB, Daratumumab, Bortezomib, Melphalan, and Prednisone

### Blood daratumumab level after switching to subcutaneous formulation

**Fig. 2:**
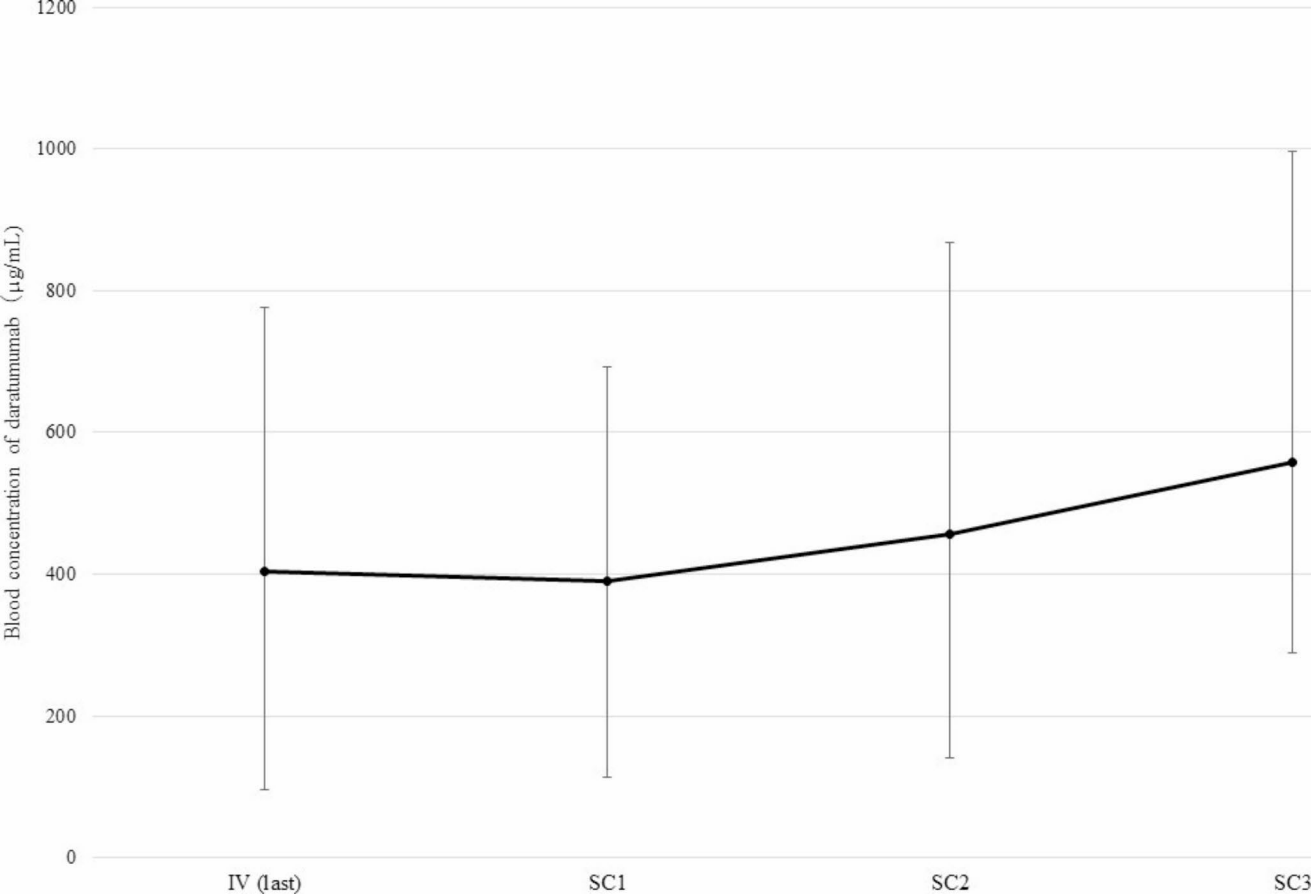
Changes in the blood concentration of daratumumab after switching from intravenous to subcutaneous formulation. The Friedman test showed a significant increase in blood levels of daratumumab after the switch (p = 0.002). The results of the analysis using Bonferroni correction were as follows. IV (last) vs. SC1 (p = 1), IV (last) vs. SC2 (p = 0.293), IV (last) vs. SC3 (p = 0.035), SC1 vs. SC2 (p = 0.164), SC1 vs. SC3 (p = 0.012), SC2 vs. SC3 (p = 0.786)

The doses and blood levels of daratumumab in 10 patients after switching from IV to SC are shown in Table 2. The median dose of IV of daratumumab was 915 mg (range, 580–1200) and the SC dose was fixed at 1800 mg. The median ratio of SC dose/IV dose was 1.95 (range, 1.50–3.10). The median blood level of daratumumab at IV (last) was 403.6 μg/mL, whereas those at SC1, SC2, and SC3 were 390.6, 457.4, and 557.1 μg/mL, respectively. The Friedman test indicated that the blood concentration of daratumumab significantly increased (p = 0.002) after switching. Using Bonferroni correction, the blood levels of daratumumab at SC3 were found to be significantly higher than those at IV (last) and SC1 (Fig. 2).

**Table 2:** Blood levels and dose of daratumumab during switching from intravenous to subcutaneous formulation

	Blood levels of Daratumumab (μg/mL)								
Patients	IV (last)	SC 1	SC 2	SC 3	Type of MM	BW (kg)	Alb (g/dL)	IV dose (mg)	SC dose/ IV dose
every four weeks									
1	776.3	313.9	868.3	997.2	IgA	36.2	4.3	580	3.10
2	96.3	113.5	140.4	288.3	IgG	45.5	3.8	730	2.47
3	381.8	386.7	516.6	544.3	IgG	48.1	3.6	770	2.34
4	502.9	408.2	458.4	672.4	IgG	55.9	3.7	900	2.00
5	319.4	394.5	288.7	413.3	IgG	61.3	3.8	980	1.84
6	425.3	432.2	456.3	569.9	IgG	62.4	4.0	1000	1.80
7	322.6	376.7	439.6	425.7	IgG	68.1	4.1	1100	1.64
8	246.4	215.2	233.9	290.4	IgA	77.1	4.1	1200	1.50
every two weeks									
1	751.5	577.2	808.3	675.8	IgA	46.9	3.8	750	2.40
2	587.1	692.1	832.1	782.3	BJP	58.0	3.9	930	1.94
Median	403.6	390.6	457.4	557.1		57.0	3.9	915	1.95
Max	776.3	692.1	868.3	997.2		77.1	4.3	1200	3.10
Min	96.34	113.5	140.4	288.3		36.2	3.6	580	1.50

IV, intravenous injection; SC, subcutaneous injection; MM, multiple myeloma; BW, body weight; BJP, Bence-Jones protein

### Safety evaluation after switching from intravenous administration to subcutaneous formulation of daratumumab

Newly developed adverse events during six months after switching from IV to SC are shown in Table 3. Only one new patient developed grade 3 neutropenia. Injection site reactions developed in six patients (60.0%). However, all cases were of grade 1–2, and no additional treatment was required. None of the patients developed IR. One patient discontinued SC of daratumumab at five months because of disease progression, but the other nine patients continued the treatment for at least six months.

**Table 3:** New adverse events after switching to subcutaneous injections for six months

Adverse events	Safety evaluation for six months after SC	
	Number of patients (%)	
	Any grade	Grade 3/4
A) Hematological related AEs		
Anemia	0 (0%)	0 (0%)
Neutropenia	3 (30.0%)	1 (10.0%)
Thrombocytopenia	0 (0%)	0 (0%)
B) Gastrointestinal disorders		
Constipation	3 (30.0%)	0 (0%)
Diarrhea	3 (30.0%)	0 (0%)
Liver impairment	0 (0%)	0 (0%)
Nausea	0 (0%)	0 (0%)
Vomiting	0 (0%)	0 (0%)
C) Other		
Any infection	0 (0%)	0 (0%)
Back pain	0 (0%)	0 (0%)
Fatigue	2 (20.0%)	0 (0%)
Headache	1 (10.0%)	0 (0%)
Infusion reaction	0 (0%)	0 (0%)
Injection site reaction	6 (60.0%)	0 (0%)
Insomnia	2 (20.0%)	0 (0%)
Peripheral neuropathy	2 (20.0%)	0 (0%)
Rash	2 (20.0%)	0 (0%)
Renal impairment	1 (10.0%)	0 (0%)

## Discussion

This is the first report regarding the safety and blood levels of daratumumab after switching from IV to SC in patients with MM. The levels of daratumumab in the blood significantly increased after switching from IV to SC in the study population. During the safety evaluation for six months, newly developed grade 3 adverse events were not observed, except in one case of neutropenia. These findings indicate that SC of daratumumab is tolerable after switching from IV, and given its shorter administration time and lower IR rate, it would prove to be useful for the treatment of MM.

In this study, the median ratio of the SC/IV dose was approximately 2; however, the median ratio of the blood levels of SC/IV was only 1.4. This relationship between the dosage and blood levels may be explained by the bioavailability of SC. The bioavailability of SC has been reported to be approximately 70% [10]. Thus, parts of the administered daratumumab could have been metabolized before entering the blood circulation. As a result, the blood concentration of daratumumab might not have increased significantly despite the doublet dose.

In addition, a high inter-individual variability in the blood levels of daratumumab was observed in the assessed patients. Serum albumin levels, body weight, type of MM, and sex are known covariates of daratumumab PK parameters [10, 11]. The blood levels of daratumumab may have varied because of the differences in these covariates; the variability was much greater than the changes in the blood levels of daratumumab after switching from IV to SC.

The incidence of injection site reactions was 60% in this study; however, these were mild grade 1–2 adverse events in all cases, did not require discontinuation or additional treatment, and were resolved by the time of the next administration. In contrast, IR did not occur after switching to SC of daratumumab despite the increase in the dose. The only new serious adverse event of grade 3 or higher during six months of study period was one case of neutropenia. Overall, SC of daratumumab was tolerable by all 10 patients assessed in this study. Another study analyzing the safety after switching to SC of daratumumab further reported that this switching was safe in patients with transplant-ineligible newly diagnosed MM [12].

This study has several limitations. First, this was a small single-center study focusing on the safety and blood levels of daratumumab after switching from IV to SC, and other patients may show a different trend. Second, although safety was evaluated for six months, blood levels of daratumumab were only evaluated until two SC doses. Long-term changes in the blood levels of daratumumab upon administration of SC were not analyzed in this study. Third, the frequency of adverse events after switching the route of administration may not have been adequately evaluated due to the small number of patients involved. Fourth, in this study, we were concerned about an increase in adverse events, including myelosuppression, in patients with low body weight, but the number of patients was limited and we were not able to fully examine low body weight and obese patients.

In conclusion, the administration of daratumumab as SC has many benefits for patients, as it reduces the administration time and IR rate. Although fixed doses led to increased blood levels of daratumumab, the changes were minor compared to the inter-individual variability. Further, the adverse effects of SC of daratumumab were generally controllable. This suggests that switching from IV to SC of daratumumab is safe and reasonable for patients with MM.

## Data Availability

All data generated or analyzed during this study are included in this published article and its supplementary information files.
